# Heavy metals bioaccumulation in Berseem (*Trifolium alexandrinum*) cultivated in areas under intensive agriculture, Punjab, India

**DOI:** 10.1186/s40064-016-1777-5

**Published:** 2016-02-25

**Authors:** Sandip Singh Bhatti, Vasudha Sambyal, Avinash Kaur Nagpal

**Affiliations:** Department of Botanical and Environmental Sciences, Guru Nanak Dev University, Amritsar, India; Department of Human Genetics, Guru Nanak Dev University, Amritsar, India

**Keywords:** Berseem, Heavy metals, Maximum permissible limits, Bioaccumulation factor

## Abstract

Berseem (*Trifolium alexandrinum*) is one of the main fodder crops of Punjab, India. But due to the heavy metal contamination of agricultural soils by anthropogenic activities, there is rise in metal bioaccumulation in crops like Berseem. In addition to human influence, heavy metal contents in soil are highly dependent on soil characteristics also. Therefore a study was conducted in areas having intensive agricultural practices to analyze physico-chemical characteristics of soils under Berseem cultivation and heavy metal bioaccumulation in Berseem. The studied soils were alkaline, sandy in texture and low in soil organic matter. Among the studied heavy metals (Cr, Cu, Cd, Co and Pb) in soil and Berseem, Cr content in Berseem was found to be above maximum permissible limits. Soil to Berseem metal bioaccmulation factor (BAF) was above 1 for Cr, Cu, Cd and Co in many samples and highest BAF was found for Co (4.625). Hence it can be concluded that Berseem from studied areas was unsafe for animal consumption.

## Background

Punjab is one of the most fertile states of India and agriculture in addition to livestock rearing is the main source of income of people of this region. Berseem (*Trifolium alexandrinum*) is the main fodder crop of Rabi season in Punjab. Due to presence of perennial rivers Beas and Sutlej and abundant groundwater resources intensive agriculture is practised in Punjab throughout the year. Berseem is a leguminous crop belonging to family Fabaceae. It is fast growing, high biomass yielding fodder crop and is highly liked by the animals (Ali et al. 2012; Bhat 2013). But rise in the level of environmental pollutants in Berseem due to uncontrolled use of agrochemicals and polluted irrigation water is a serious cause of concern for livestock production systems (Rajaganpathy et al. 2011).

Heavy metals are one of the main pollutants which affect the plants and animals throughout globe. Food and fodder crops raised on metal contaminated soils have the tendency to accumulate excessive amounts of heavy metals, which poses severe risk to human and animal health (Rattan et al. 2005; Kulhari et al. 2013). Heavy metal contents in fodder crops are dependent on soil, climatic factors, agrochemical application, irrigation water quality, plant growth rates and plant parts (Ahmad et al. 2011). The heavy metal contents in agricultural soil are affected by cropping practices and soil properties (Dheri et al. 2007; Endalamaw and Chandravanshi 2015). In the areas which are situated on the banks of rivers there is an added risk of heavy metal contamination in soils due to rise in pollution in rivers, because in these areas river water is also used for irrigation. Although heavy metals like Co, Cr and Cu are essential for plant and animal metabolism, at levels above maximum permissible limits they disrupts the normal functioning of organisms (Ali et al. 2012; Jolly et al. 2013). Cd and Pb are known to be highly toxic and carcinogenic for animals and humans (Rajaganpathy et al. 2011). Since heavy metal contents in soil are highly affected by soil characteristics and heavy metal bioaccumulation in fodder crops is a major route of heavy metal entry into livestock systems, a study was conducted in intensively cultivated areas situated on banks of rivers Sutlej and Beas to assess physcio-chemical characteristics of soil under Berseem cultivation and heavy metal bioaccumulation in Berseem.

## Results and discussion

### Soil physico-chemical characteristics

The physico-chemical characteristics of the studied soils are given in Table 1. The Pearson’s correlation matrix of physico-chemical properties and heavy metals in studied soils are given in Table 2. The soils were found to be slightly alkaline in nature and the pH ranged from 7.38 to 7.99. Soil conductivity which indicates the salinity was maximum at site I (0.723 mS/cm) and minimum at site II (0.307 mS/cm). The observed soils were found to be sandy in texture with sand contents ranging from 3.15 (Site I) to 5.33 % (Site II). The sandy texture of soils is mainly due to settling of sandy river alluvium deposited for thousands of years by rivers Sutlej and Beas. The soil organic matter (SOM) which is one of the most important indicators of soil health (Rattan et al. 2005) was found to be near desertification levels (1.83–2.91 %) in the present study. The main reason for such low levels of SOM is the poor silt and clay contents of soils (Sollins et al. 1996). Significant positive correlation was observed between soil pH and SOM, which was in line with the observations of Aciego Pietri and Brookes (2008), but was contradictory to the trend observed by Yali et al. (2012). The carbonate content (CaCO_3_) ranged from 5.07 to 9.04 % revealing the calcareous nature of the studied soils. The calcium (Ca) and magnesium (Mg) contents in soils ranged from 0.49 to 1.00 meq/100 g and 0.73–1.74 meq/100 g respectively. Ca and Mg are secondary nutrients for plants and abundantly available in soils to meet plant requirements. But excessive amount of Ca in soils sometimes causes deficiency of other plant nutrients (Troeh and Thompson 2005), which is evident from the negative correlation of Ca with sodium (Na), potassium (K) and nitrogen (N) (Table 2). The major soil nutrients nitrogen (N), phosphorous (P) and potassium (K) ranged from 114.33 to 665.15, 49.99–384.91 and 1052.83–1559.67 mg/kg respectively. The soil nutrients (Na, N and K) were found to be negatively correlated to sand and positively correlated to clay (Table 2), which can be due to high affinity of clay to soil nutrients (Boluda et al. 2011). The main anthropogenic sources of soil nutrients in the studied soils are NPK fertilizers, which are extensively used in the studied area. The NPK fertilizers are favored by the high pH of soils (Gil et al. 2004), which is evident from the positive correlation of pH with N, P and K (Table 2). The levels of soil physico-chemical parameters in the present study were similar to the levels observed in other parts of Punjab (Dheri et al. 2007; Katnoria et al. 2011).

**Table 1:** Physico-chemical characteristics of soil under Berseem cultivation

Physico-chemical properties	Sites			
	Site I	Site II	Site III	Site IV
pH	7.99 ± 0.25	7.60 ± 0.71	7.38 ± 0.94	7.88 ± 0.74
Conductivity (mS/cm)	0.723 ± 0.066	0.307 ± 0.038	0.617 ± 0.089	0.440 ± 0.057
Sand (%)	88.33 ± 3.36	76.67 ± 9.43	90.33 ± 3.32	85.33 ± 5.31
Silt (%)	8.33 ± 1.64	18.0 ± 2.14	6.33 ± 1.66	10.33 ± 2.31
Clay (%)	3.15 ± 0.66	5.33 ± 1.33	3.66 ± 0.97	4.33 ± 1.01
SOM (%)	2.91 ± 0.89	1.83 ± 0.31	1.88 ± 0.35	2.68 ± 0.21
CaCO_3_ (%)	5.07 ± 0.33	8.61 ± 0.97	9.04 ± 1.16	5.70 ± 0.78
Ca (meq/100 g soil)	0.87 ± 0.13	0.67 ± 0.06	1.00 ± 0.18	0.49 ± 0.07
Mg (meq/100 g soil)	1.74 ± 0.17	0.94 ± 0.14	0.73 ± 0.09	0.79 ± 0.13
Na (mg/kg)	354.33 ± 11.57	403.50 ± 35.42	404.16 ± 17.76	1073.67 ± 98.92
N (mg/kg)	319.67 ± 76.56	665.15 ± 140.93	114.33 ± 16.65	254.33 ± 42.77
P (mg/kg)	384.91 ± 54.46	49.99 ± 11.57	190.44 ± 27.01	98.15 ± 14.27
K (mg/kg)	1052.83 ± 81.79	1315.72 ± 71.98	1183.33 ± 87.66	1559.67 ± 101.03

*SOM*, soil organic matter, *CaCO*_*3*_ Carbonates, *Ca* calcium, *Mg* magnesium, *Na* sodium, *N* Kjehldal nitrogen, *P* available phosphorous, *K* potassium

**Table 2:** Pearson’s correlation matrix of physico-chemical properties and heavy metal contents of soil

	pH	Cond.	Sand	Silt	Clay	SOM	Ca	Mg	Na	K	N	P	CaCO_3_	Cr	Cu	Cd	Co
Cond.	0.23																
Sand	0.03	0.88**															
Silt	−0.02	−0.85**	−0.99**														
Clay	−0.05	−0.83**	−0.85**	0.79**													
SOM	0.62*	0.39	0.32	−0.32	−0.24												
Ca	−0.39	0.51	0.44	−0.42	−0.47	−0.08											
Mg	0.64*	0.53	0.15	−0.11	−0.34	0.39	0.17										
Na	0.33	−0.34	−0.01	−0.04	0.18	0.30	−0.60*	0.89**									
K	0.06	−0.71**	−0.38	0.33	0.52	0.01	−0.65*	−0.60*	0.89**								
N	0.17	−0.61**	−0.86**	0.88**	0.61*	−0.37	−0.33	0.19	−0.21	0.08							
P	0.43	0.90**	0.64*	−0.60*	−0.68*	0.49	0.43	0.72**	−0.42	0.75**	−0.33						
CaCO_3_	−0.92**	−0.38	−0.26	0.25	0.27	−0.77*	0.23	−0.57	−0.40	−0.04	0.12	−0.51					
Cr	0.13	0.13	−0.13	0.09	0.28	−0.24	0.00	0.24	−0.43	−0.42	0.30	0.20	0.01				
Cu	−0.17	0.66	0.80**	−0.77**	−0.83**	0.43	0.54	0.02	−0.04	−0.30	−0.81**	0.48	−0.13	−0.52			
Cd	−0.18	0.14	−0.19	0.20	0.10	−0.49	0.07	0.27	−0.77**	−0.64*	0.33	0.25	0.34	0.69*	−0.35		
Co	0.54	−0.23	0.02	−0.05	0.14	0.45	−0.65*	−0.20	0.97**	0.80**	−0.17	−0.25	−0.59*	−0.38	−0.04	−0.73**	
Pb	−0.10	−0.54	−0.12	0.08	0.29	−0.02	−0.59*	−0.70*	0.89**	0.93**	−0.20	−0.67*	0.03	−0.49	−0.05	−0.65*	0.77**

* Correlation is significant at the 0.05 level (2-tailed)

** Correlation is significant at the 0.01 level (2-tailed)

### Heavy metal contents in soil

**Fig. 1:**
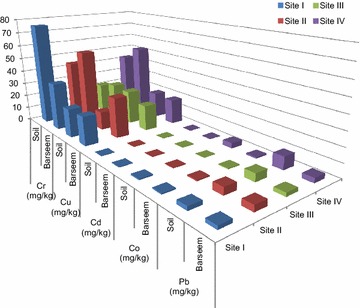
Heavy metal contents in soil and Berseem samples of Punjab, India

Figure 1 shows the contents of different heavy metals (Cr, Cu, Cd, Co and Pb) studied in soil and Berseem. Among these metals, Cr was found to be highest in all soil samples. Maximum amount of Cr was observed at Site I (75.70 mg/kg) and minimum level was observed at Site III (21.37 mg/kg). Cu, Cd and Co ranged from 13.67 to 25.0, 0.15–0.37 and 0.13–3.83 mg/kg respectively. Pb contents ranged from 2.83 mg/kg (Site I) to 9.17 mg/kg (Site IV). The main sources of heavy metals in the soil samples are parent rock material, polluted irrigation water and various agrochemicals (fertilizers, pesticides, weedicides etc.) used for cultivation. The studied sites are located on the banks of rivers Sutlej and Beas which have been shown to be highly polluted with industrial effluents, sewage discharge and agricultural runoffs (Kaur et al. 2014a, b). The groundwater is also contaminated due to leaching of pollutants from surface water and upper soils layers. Some earlier studies have reported dangerous levels of heavy metals in the surface and groundwater of neighbouring regions of the sampled areas and other parts of Punjab (Kumar et al. 2007; Bhalla et al. 2011; Singh et al. 2013; Kaur et al. 2014a, b; Shrivastava 2014). Sewage discharge and effluents originating from various industries such as electroplating, dying, leather tanning, alloys, paints etc. situated upstream of these sites, especially in the neighbouring cities Ludhiana, Jalandhar, Kapurthala etc. are the main source of contamination of surface and ground water in these areas. The polluted irrigation water acts as a major source of heavy metals in the analyzed soil samples. Secondly, excessive use of NPK fertilizers and other agrochemicals further contaminate the soil with heavy metals. Various NPK fertilizers act as source of heavy metals such as Cd, As, Pb, Cr, Ni, Cu etc. The Phosphate fertilizers in particular, have substantial amounts of Cd in them (Mortvedt 1996; Milinovic et al. 2008; Savci 2012). Among the studied heavy metals in the present work Cu, Co and Cr are required in small amounts for plant metabolism. Cd and Pb are non essential and toxic elements for plants and other soil organisms (Mertz 1981), which was evident from the negative correlation observed between SOM, Pb and Cd (Table 2). Pb being a toxic metal disrupts the total chlorophyll content of plants and decreases root expansion by restricting the cell division and cell elongation (Eun et al. 2000; McDermott et al. 2011). Thus it is a restricting factor for SOM.

The heavy metal contents in soil are also dependent on soil physico-chemical properties, which affect the mobility, availability and ecotoxicological risks of heavy metals (Boluda et al. 2011). The heavy metal contents in soil samples observed in the present analysis are below the maximum permissible limits (Table 3) despite the extensive agricultural practices and polluted irrigation water, which can be attributed to sandy texture of soil. Due to high sand contents in soil the metals can leach to lower soil layers (Boluda et al. 2011), which was evident from the negative correlation between heavy metals and sand (Table 2). The contents of Pb and Cd observed in the present study were lower and Cr contents were higher than the earlier works done by Dheri et al. (2007) and Chahal et al. (2014).

**Table 3:** National and international maximum permissible limits of heavy metals for soil and fodder

	Chromium (mg/kg)	Copper (mg/kg)	Cadmium (mg/kg)	Cobalt (mg/kg)	Lead (mg/kg)
*Maximum permissible limits for soil*					
Indian (Awashthi 2000)*	–	135–270	3–6	–	250–500
European Union (2002)	150	140	3.0	–	300
*Maximum permissible limits for fodder*					
CERSPC (2009)	10.0	–	0.5	–	5.0

* Prevention of Food Adulteration Act No. 37 of 1954. Central and State Rules as Amended for 1999 (Sharma et al. 2006; Singh et al. 2010)

### Heavy metal contents in Berseem

Similar to the results observed in soil, Cr contents were highest in Berseem in comparison to the other metals (Fig. 1). Maximum amount of Cr was found in Berseem samples from Site II (43.43 mg/kg) and minimum content was observed at Site III (25.92 mg/kg). Cr contents were alarmingly higher than the maximum permissible limits for fodder set by Tolerance Limit of Heavy Metals for Feed of China (CERSPC 2009) in all the samples (Table 3). Cr content above the permissible limits can cause deleterious effects on plant physiological processes such as photosynthesis and respiration (Ahmad et al. 2013). Although trace amounts of Cr is required for glucose metabolism in animals, but Cr overdose can cause liver necrosis, nephrites, gastrointestinal irritation and ulcers (coetaneous, nasal and mucous membrane) (Edwards and Gregory 1991; ATSDR 2000). The contents of Cu and Cd ranged from 13.67 to 21.35 and 0.15–0.37 mg/kg respectively. Cu is an important element for plant nutrition but Cd despite being a non-essential element in plants gets highly accumulated in plants (Nadian 2004). Excess amount of Cd in animal fodder can result in renal tubule damage, cardiovascular diseases, cancer, ostomalacia and deleterious effects on calcium, phosphorous and bone metabolism (Katole et al. 2013). Co contents were found to be maximum at Site IV (1.07 mg/kg) and minimum at Site III (0.62 mg/kg) which can be due to minimum and maximum levels of Co at the same sites in soil samples. Pb contents ranged from 2.5 to 4.33 mg/kg which is found to be close to the maximum permissible limit of 5 mg/kg (Table 3) at Site II (4.33 mg/kg). Pb at high levels decreases root expansion by restricting cell division and elongation in plants. Excess amounts of Pb in animal fodder can cause acute or chronic poisoning in animals leading to decreased haemoglobin synthesis, neurobehavioral impairment, peripheral neuropathy reproductive effects and neurotoxic malfunctioning in infants (Allcroft and Blaxter 1950). In Punjab lower levels of Cr and Cd and higher level of Pb in Berseem were observed by Dheri et al. (2007). Very high levels of Pb (102–383 mg/kg) were observed in fodder feed to animals in industrial areas of Punjab by Sidhu et al. (1994) which resulted in Pb toxicity (blood) in bovine animals. Heavy metal toxicity (Pb, Cd, Cr, Cu, Mn etc.) in animals due to contaminated fodder has been observed by many researchers in other parts of India also (Dey et al. 2011; Gowda et al. 2003; Raj et al. 2006). Therefore the Berseem fodder from the studied areas was contaminated with heavy metals due to combined effect of several anthropogenic factors (such as pollution of irrigation water, excessive use of agrochemicals, etc.) and is a serious cause of concern for animal health.

### Metal bioaccumulation factor (BAF)

**Fig. 2:**
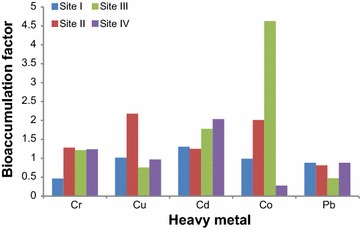
Soil to Berseem heavy metal bioaccumulation factor (BAF)

Heavy metal bioaccumulation factor (BAF) values above 1 were observed for Cr, Cu, Cd and Co (Fig. 2) in many samples, which showed high level of metal bioaccumulation in Berseem. Highest value of BAF was observed for Co (4.625) at Site III. Studies on metal uptake by plants have revealed that heavy metals are passively transported from roots to shoots via xylem vessels and are preferably accumulated in areas with high transpiration rates (Tamoutsidis et al. 2009). Since the most important parts in Berseem from commercial point are leaves and shoot, heavy metal accumulation in these parts poses severe risk to animals. This problem is further aggravated by the high phytoremediation potential of Berseem (Ali et al. 2012; Bhat 2013).

## Conclusions

The present study revealed that the areas studied have alkaline soils with sandy texture, calcareous nature and low SOM. The Cu, Cd, Co and Pb contents in studied soil and Berseem samples were lower than the maximum permissible limits. But Cr contents in Berseem were higher than the maximum permissible limits for fodder (CERSPC 2009). BAF was found to be above 1 for Cr, Cu, Cd and Pb for many Berseem samples. Therefore it can be concluded that there is significant bioaccumulation of heavy metals in Berseem grown in these intensively cultivated areas which can be due to synergistic effect of various anthropogenic factors such as irrigation water contamination, indiscriminate use of agrochemicals, industrial activities, etc. and thus it is unsafe for animal consumption.

## Methods

### Study area

**Fig. 3:**
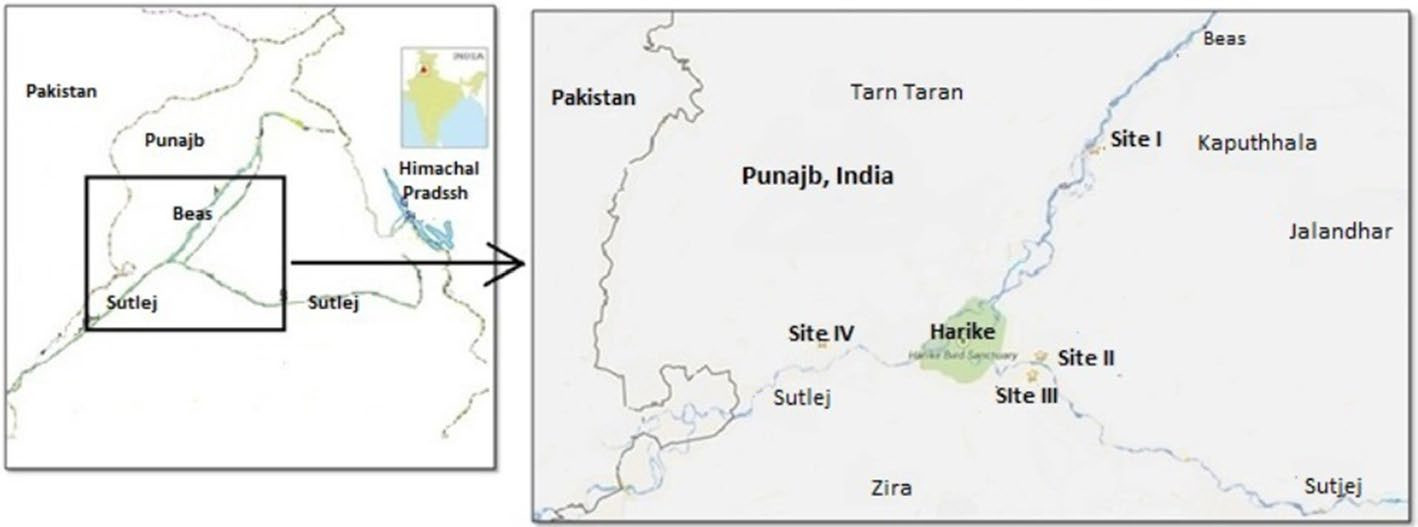
Soil and Berseem sampling sites in Punjab, India

The state of Punjab (Lat. 29°30–32°32′N and Long. 73°55 and 76°50′E) is located in the north-western part of India bordering Pakistan. The annual rainfall in Punjab is 435.6 mm and has a continental, semiarid to subhumid climate with two main crop seasons Kharif (fall) and Rabi (spring). Four sites were selected for sampling, which are situated on the banks of rivers Beas and Sutlej. The geographical coordinates of sites are given in Table 4 and Fig. 3 shows the map of studied area. The upstream regions of the sites chosen for sampling are receiving effluents from various industries (electroplating, dying, leather tanning, alloys, paints etc.) and sewage. The main occupation in the study area is agriculture which includes extensive use of agrochemicals (fertilizers, pesticides and weedicides).

**Table 4:** List of sampling sites with their districts, adjoining river, geographical coordinates

S. No.	Sites	Village	District	Adjoining river	Coordinates
1.	Site I	Rajewal	Kapurthala	Beas	N31°22′59.2″ E075º10′47.6″
2.	Site II	Yousufpur	Kapurthala	Sutlej	N31°08′47.1″ E075º06′22.4″
3.	Site III	Tibbi Taiba	Firozpur	Sutlej	N31°07′23.1″ E075º05′42.4″
4.	Site IV	Doomniwala	Tarn Taran	Sutlej	N31°09′43.1″ E074º48′50.0″

### Sampling and preparation

Soil sampling was done during the period of March–April 2013. From each site composite soil samples in triplicates were collected from fields under Berseem cultivation. At least five subsamples of soil were pooled to form a composite sample. Soil samples were taken from depths of 0–15 cm. Composite samples of Berseem (Shoots) were collected in triplicates from corresponding soil sampling fields from each site for heavy metal analysis. All soil and Berseem samples were stored in clean polythene bags and were brought to the laboratory. The soil samples were air-dried, ground and passed through 2 mm sieve for physico-chemical and heavy metal analysis. The Berseem shoot samples were washed with deionised water, oven dried at 70 °C and then grounded to fine powder with pestle mortar. Similar methods have been used for collection and preservation of soil and plant samples by researchers earlier also (Yao et al. 2010; Bermudez et al. 2011; Rodriguez Martin et al. 2013; Liu et al. 2013).

### Physico-chemical analysis

The soil pH and conductivity were determined in 1:5 soil:water suspension using HM digital meter-COM-100 (New Delhi, India) and Equip-tronics EQ-614-A (Mumbai, India), respectively. The soil suspension was prepared by mixing soil and water in desired proportion (1:5) and the mixture was shaken for 2 h and the supernatant was filtered and used for measurement. Soil texture and organic carbon content was determined by Hydrometer method (Jacob and Clarke 2002) and Walkley–Black wet oxidation method (Nelson and Sommers 1982) respectively. A factor of 1.72 was multiplied with organic carbon content to determine SOM. EDTA titration method was used for measuring calcium (Ca) and magnesium (Mg) (Lanyon and Heald 1982), acid neutralization method for CaCO_3_ (Hesse 1971) and potassium (K) and sodium (Na) were measured by using a Systronics Flame Photometer-128, after digesting the samples in a diacid mixture (HClO4/HNO3 in a 4:1 ratio) (Bhat et al. 2014). Total nitrogen (N) was determined by Kjeldahl method (Bremner and Mulvaney 1982) and available phosphorous (P) by sodium bicarbonate extraction method using Spectrophotometer-2202 (Ahmedabad, Gujarat, India) (Olsen et al. 1954).

### Heavy metal analysis

For heavy metal (Cr, Cu, Cd, Co and Pb) determination one gram of soil was digested with 15 mL of aqua regia (HNO_3_: HCl in 3:1 ratio) and 1 g Berseem shoot sample with 15 mL of triacid mixture (HNO_3_:H_2_SO_4_:HClO_4_ in 5:1:1 ratio) at 80 °C till a transparent solution was obtained (Allen et al. 1986). The digested samples were filtered and diluted with de-ionized water up to 50 mL and analyzed for the metals viz. Chromium (Cr), copper (Cu), cadmium (Cd) cobalt (Co) and lead (Pb) by flame atomic absorption spectrophotometer (AAS) (Agilent 240 FS AA model). Properly washed glassware, double distilled water and analytical grade reagents were used throughout the study. The standard solutions of selected heavy metals were procured from Agilent (1000 mg/L) and were used to make solutions of varying concentrations by dilution of the standards. After every ten sample readings, the standards were run to assure the working of machine with 95 % accuracy (Arora et al. 2008).

### Metal bioaccumulation factor (BAF)

Heavy metal accumulation of soil and Berseem were calculated on the basis of dry weight. The metal bioaccumulation factor is a ratio of heavy metal concentration of crop to soil (Zhuang et al. 2013) and was calculated as follows:

1 $$BAF = C_{plant} /C_{soil}$$

where *C*_plant_ and *C*_soil_ are the concentrations of heavy metal in Berseem and soil, respectively, on a dry weight basis.

### Statistical analysis

The analysis of physico-chemical characteristics and heavy metal content of soil and Berseem samples was done in triplicates and the data is presented as mean ± standard error. Pearson correlation coefficients were calculated to analyze the correlation between physico-chemical properties and heavy metals in soil. Statistical analysis was done with the help of IBM SPSS version 16.0 (Chicago, USA) and Microsoft excel computer software programs.

## Abbreviations

BAF: bioaccumulation factor
Cr: chromium
Cu: copper
Cd: cadmium
Co: cobalt
Pb: lead

## References

[CR1] Aciego Pietri JC, Brookes PC (2008). Relationships between soil pH and microbial properties in a UK arable soil. Soil Biol Biochem.

[CR2] Ahmad K, Ibrahim M, Khan ZI, Rizwan Y, Ejaz A, Fardsous A, Gondal S, Lee DJ, Al-Yemeni M (2011). Effect of sewage water on mineral nutritive potential of six fodder species grown under semiarid conditions. Saudi J Biol Sci.

[CR3] Ahmad K, Shaheen M, Khan ZI, Bashir H (2013). Heavy metals contamination of soil and fodder: a possible risk to livestock. Sci Tech Dev.

[CR4] Ali H, Naseer M, Sajad MA (2012). Phytoremediation of heavy metals by *Trifolium alexandrinum*. Int J Environ Sci.

[CR5] Allcroft R, Blaxter KL (1950). Lead as a nutritional hazard to farm livestock. The toxicity of lead to cattle and sheep and an evaluation of the lead hazard under farm conditions. J Comp Pathol.

[CR6] Allen SE, Grimshaw HM, Rowland AP, Moore PD, Chapman SB (1986). Chemical analysis. Methods in plant ecology.

[CR7] Arora M, Kiran B, Rani S, Rani A, Kaur B, Mittal N (2008). Heavy metal accumulation in vegetables irrigated with water from different sources. Food Chem.

[CR8] ATSDR (2000). Toxicological profile of chromium.

[CR9] Awashthi SK (Ed.) (2000). Prevention of Food Adulteration Act No. 37 of 1954. Central and State Rules as Amended for 1999, Ashoka Law House, New Delhi

[CR10] Bermudez GMA, Jasan R, Pla R, Pignata ML (2011). Heavy metal and trace element concentrations in wheat grains: assessment of potential non-carcinogenic health hazard through their consumption. J Hazard Mater.

[CR11] Bhalla A, Singh G, Kumar S, Shahi JS, Mehta D (2011). Elemental analysis of ground water from different regions of Punjab state (India) using EDXRF technique and the sources of water contamination. Int Conf Environ Comput Sci.

[CR12] Bhat S (2013). Phytoremidation properties and CLA content of Berseem (*Trifolium alexandrinum*). Asian J Microbiol Biotechnol Environ Sci.

[CR13] Bhat SA, Singh J, Vig AP (2014). Genotoxic assessment and optimization of pressmud with the help of exotic earthworm *Eisenia fetida*. Environ Sci Pollut Res.

[CR14] Boluda R, Roca-Perez L, Marimon L (2011). Soil plate assay: an effective method to determine ecotoxicological risks. Chemosphere.

[CR15] Bremner JM, Mulvaney CS, Page AL, Miller RH, Keeney DR (1982). Nitrogen total. Methods of soil analysis.

[CR16] Chahal V, Chand P, Nagpal A, Katnoria JK, Pakade YB (2014). Evaluation of heavy metals contamination and its genotoxicity in agricultural soil of Amritsar, Punjab, India. Int J Res Chem Environ.

[CR17] Chief Editor Room of Standard Press of China (CERSPC) (2009). Compilation of standards for feed industry.

[CR18] Dey S, Dwivedi SK, Swarup D (2011). Heavy metal contaminants in soil, water and fodder and their presence in livestock and products: a review. J Environ Sci Technol.

[CR19] Dheri GS, Brar MS, Malhi SS (2007). Heavy-metal concentration of sewage-contaminated water and its impact on underground water, soil, and crop plants in alluvial soils of northwestern India. Commun Soil Sci Plant Anal.

[CR20] Edwards WC, Gregory DG (1991). Livestock poisoning from oil field drilling fluids, muds and additives. Vet Hum Toxicol.

[CR21] Endalamaw FD, Chandravanshi BS (2015). Levels of major and trace elements in fenel (*Foeniculum vulgari* Mill.) fruits cultivated in Ethiopia. Springerplus.

[CR22] Eun SO, Youn HS, Lee Y (2000). Lead disturbs microtubule organization in root meristem of *Zea mays*. Physiol Plant.

[CR23] European Union (2002) Heavy metals in wastes, European Commission on Environmet. http://ec.europa.eu/environment/waste/studies/pdf/heavymetalsreport.pdf. Assessed 21 July 2014

[CR24] Gil C, Boluda R, Ramos J (2004). Determination and evaluation of cadmium, lead and nickel in greenhouse soils of Almería (Spain). Chemosphere.

[CR25] Gowda NKS, Malathi VS, Jash S, Roy KS (2003). Status of pollutants and trace elements in water, soil, vegetation and dairy animals in industrial area of Banglore. Indian J Dairy Sci.

[CR26] Hesse PR (1971). A textbook of soil chemical analysis.

[CR27] Jacob H, Clarke G (2002). Part 4, Physical Method.

[CR28] Jolly Y, Islam A, Akbar S (2013). Transfer of metals from soil to vegetables and possible health risk assessment. Springerplus.

[CR29] Katnoria JK, Arora S, Bhardwaj R, Nagpal A (2011). Evaluation of genotoxic potential of industrial waste contaminated soil extracts of Amritsar, India. J Environ Biol.

[CR30] Katole SB, Kumar P, Patil RD (2013). Environmental pollutants and livestock health: a review. Vet Res Int.

[CR31] Kaur J, Chaudhary A, Kaur R (2014). Assessment of mutagenic, genotoxic and cytotoxic potential of water samples of Harike wetland: a Ramsar site in India using different ex vivo biological systems. Ecotoxicology.

[CR32] Kaur T, Sharma K, Sinha AK, Sharma K, Sinha AK (2014). Industrial pollution in the sub-soil water and its health effects: a preliminary study around Buddha Nullah, Punjab. Human ecology in an era of globalization and urbanization.

[CR33] Kulhari A, Sheorayan A, Bajar S, Sarkar S, Chaudhury A, Kalia RK (2013). Investigation of heavy metals in frequently utilized medicinal plants collected from environmentally diverse locations of north western India. Springerplus.

[CR34] Kumar M, Kumari K, Ramanathan AL, Saxena R (2007). A comparative evaluation of groundwater suitability for irrigation and drinking purposes in two intensively cultivated districts of Punjab, India. Environ Geol.

[CR35] Lanyon LE, Heald WR (1982) Magnesium, calcium, strontium and barium. Agronomy No. 9

[CR36] Liu X, Song Q, Tang Y, Li W, Xu J, Wu J, Wang F, Brookes PC (2013). Human health risk assessment of heavy metals in soil-vegetable system: a multi-medium analysis. Sci Total Environ.

[CR37] McDermott S, Wu J, Cai B, Lawson A, Marjorie Aelion C (2011). Probability of intellectual disability is associated with soil concentrations of arsenic and lead. Chemosphere.

[CR38] Mertz W (1981). The essential trace elements. Science.

[CR39] Milinović J, Lukić V, Nikolic-Mandić S, Stojanović D (2008). Concentrations of heavy metals in NPK fertilizers imported in Serbia. Pestic Phytomed.

[CR40] Mortvedt JJ (1996). Heavy metal contaminants in inorganic and organic fertilizers. Fertil Res.

[CR41] Nadian H (2004) Cd and Mn uptake and bioaccumulation in *Trifolium alexandrinum* L.: interaction with mycorrhizal colonization. In: Proceedings of the fourth international Iran and Russia conference, pp 595–601

[CR42] Nelson DW, Sommers LE, Page AL (1982). Total carbon and organic matter. Methods of soil analysis, part 2.

[CR43] Olsen SR, Cole CV, Watanabe FS, Dean LA (1954) Estimation of available phosphorous in soils by extraction with sodium bicarbonate. USDA Circ. No. 939. Washington, DC, US Dept. Agric

[CR44] Raj BG, Patnaik MC, Babu SP, Kalakumar B, Singh MV, Shylaja J (2006). Heavy metal contaminats in water-soil-plant-animal continuum due to pollution of Musi river around Hyderabad in India. Indian J Anim Sci.

[CR45] Rajaganpathy V, Xavier F, Sreekumar D, Mandal PK (2011). Heavy metal contamination in soil, water and fodder and their presence in livestock and products: a review. J Environ Sci Technol.

[CR46] Rattan RK, Datta SP, Chhonkar PK, Suribabu K, Singh AK (2005). Long-term impact of irrigation with sewage effluents on heavy metal content in soils, crops and groundwater: a case study. Agric Ecosyst Environ.

[CR47] Rodriguez Martin JA, Ramos-Miras JJ, Boluda R, Gil C (2013). Spatial relations of heavy metals in arable and greenhouse soils of a Mediterranean environment region (Spain). Geoderma.

[CR48] Savci S (2012). An agricultural pollutant: chemical fertilizer. Int J Environ Sci Dev.

[CR49] Sharma RK, Agarwal M, Marshall F (2006). Heavy metal contamination in vegetables grown in wastewater irrigated areas of Varanasi, India. Bull Environ Contam Toxicol.

[CR50] Shrivastava BK (2014). Elevated uranium and toxic elements concentration in groundwater in Punjab state of India: extent of the problem and risk due to consumption of unsafe drinking water. Water Qual Expo Health.

[CR51] Sidhu PK, SandhuBS Singh J, Kwatra MS (1994). Lead toxicosisin bovine due to industrial pollution in Punjab. Indian J Anim Sci.

[CR52] Singh A, Sharma RK, Agrawal M, Marshall FM (2010). Health risk assessment of heavy metals via dietary intake of foodstuffs from the wastewater irrigated site of a dry tropical area of India. Food Chem Toxicol.

[CR53] Singh CK, Rina K, Singh RP, Mukherjee S (2013). Geochemical characterization and heavy metal contamination of groundwater in Sutlej River Basin. Environ Earth Sci.

[CR54] Sollins P, Homann P, Caldwell BA (1996). Stabilization and destabilization of soil organic matter: mechanisms and controls. Geoderma.

[CR55] Tamoutsidis E, Lazaridou M, Papadopoulos I, Spanos T, Papathanasiou F, Tamoutsidou M, Mitlianga P, Vasiliou G (2009). The effect of treated urban wastewater on soil properties, plant tissue composition and biomass productivity in berseem clover and corn. J Food Agric Environ.

[CR56] Troeh FR, Thompson LM (2005). Soil and soil fertility.

[CR57] Yali W, Zhonghao Z, Gangcai L (2012). Physico-chemical properties and enzyme activities of the arable soils in Lhasa, Tibet, China. J Mt Sci.

[CR58] Yao F, Changcun L, Jianjun M, Tingcheng Z (2010). Effects of plant types on physico-chemical properties of reclaimed mining soil in inner Mangolia, China. Chin Geogr Sci.

[CR59] Zhuang P, Li Z, Zou B, Xia H, Wang G (2013). Heavy metal contamination in soil and soyabean near the Dabaoshan mine, South China. Pedosphere.

